# The distribution of child physicians and early academic achievement

**DOI:** 10.1111/1475-6773.14188

**Published:** 2023-06-07

**Authors:** Jessica Drescher, Benjamin W. Domingue

**Affiliations:** ^1^ Center for Education Policy Analysis Stanford University Graduate School of Education Stanford California USA; ^2^ Center for Population Health Sciences Stanford University School of Medicine Stanford California USA

**Keywords:** child development, education, health policy, physician supply, social determinants of health, workforce

## Abstract

**Objective:**

To describe the distribution of pediatricians and family physicians (*child physicians*) across school districts and examine the association between physician supply and third‐grade test scores.

**Data Sources and Study Setting:**

Data come from the January 2020 American Medical Association Physician Masterfile, the 2009–2013 and 2014–2018 waves of American Community Survey 5‐Year Data, and the Stanford Education Data Archive (SEDA), which uses test scores from all U.S. public schools. We use covariate data provided by SEDA to describe student populations.

**Study Design:**

This descriptive analysis constructs a physician‐to‐child‐population ratio for every school district in the country and describes the child population served by the current distribution of physicians. We fit a set of multivariable regression models to estimate the associations between district test score outcomes and district physician supply. Our model includes state fixed effects to control for unobservable state‐level factors, as well as a covariate vector of sociodemographic characteristics.

**Data Collection:**

Public data from three sources were matched by district ID.

**Principal Findings:**

Physicians are highly unequally distributed across districts: nearly 3640 (29.6%) of 12,297 districts have no child physician, which includes 49% of rural districts. Rural children of color in particular have very little access to pediatric care, and this inequality is more extreme when looking exclusively at pediatricians. Districts that have higher child physician supplies tend to have higher academic test scores in early education, independent of community socioeconomic status and racial/ethnic composition. While the national data show this positive relationship (0.012 SD, 95% CI, 0.0103–0.0127), it is most pronounced for districts in the bottom tertile of physician supply (0.163 SD, 95% CI, 0.108–0.219).

**Conclusions:**

Our study demonstrates a highly unequal distribution of child physicians in the U.S., and that children with less access to physicians have lower academic performance in early education.

**What is known on this topic**

- Early academic achievement is highly influenced by out‐of‐school factors.
- Higher physician supply is associated with better child health outcomes, including improved access to care, reduced prevalence of unnecessary hospitalizations, and lower rates of infant mortality and low birth weight.
- The child health outcomes influenced by physician supply are linked to educational success in both direct and indirect ways: e.g., lower birth weight is directly linked to reduced cognitive performance.

**What this study adds**

- This study introduces a novel, school district‐level measure of physician supply, providing the first glimpse at the national distribution of pediatricians and family physicians across school districts.
- Physician‐to‐child‐population ratios are lowest in rural districts, especially rural districts with large non‐White student populations. Nationally, the ratios are not correlated with district socioeconomic status or racial/ethnic composition.
- District‐level variation in the supply of physicians trained in pediatric care is associated with variation in third‐grade test scores and is especially pronounced in areas with low physician supply.

## 1 INTRODUCTION

Academic achievement among U.S. public school students, as measured by standardized test scores, varies widely by geography, socioeconomic status, race, and ethnicity. Many decades of education research have closely examined average test score differences among students on a national scale, starting with the 1966 Coleman Report which famously concluded that only 10%–20% of the variation in student achievement scores is attributable to schools.^1^ Recent breakthroughs in our ability to compare test score data across states, cohorts, and subjects have generated an abundance of new comparisons and improved our ability to document the ways that average achievement is closely associated with underlying social conditions.^2^ Research has consistently reported that a student's socioeconomic status (SES) is the greatest predictor of test score outcomes, and gaps between low‐SES and high‐SES students have grown larger over time.^3^, ^4^ These gaps are closely linked to growing income inequality and racial/ethnic residential segregation.^4^, ^5^, ^6^ For example, evidence shows that racial/ethnic achievement gaps vary substantially, from almost zero in some places to 1.5 standard deviations in others, and much of the variation appears to be driven by racial/ethnic differences in parental income, education, and residential patterns.^7^, ^8^ These findings shed light on the complex and interwoven ways that educational opportunities are stratified within and across U.S. communities.

Despite these advances in our understanding of how sociodemographic factors relate to test score patterns across the country, there is still much to understand about the spatial variation in test scores. How else does place shape test score outcomes? This question is particularly salient for younger students, who have less exposure to formal schooling but arrive at elementary school with systemic gaps in test score performance.^9^ Education researchers acknowledge that gaps in achievement are evident very early in life and attributable to structural inequalities in out‐of‐school factors, but our understanding of the social conditions that drive these differences is limited.^10^ One under‐explored area for examining early differences in achievement is children's local health environments. While some research has linked rates of child insurance to test score outcomes, there are many aspects of children's health that could inform our understanding of early childhood wellbeing and educational opportunity.^11^, ^12^ This paper tests whether a novel variable in the early childhood environment may be associated with patterns of early academic achievement: the local supply of pediatricians and family physicians.

The local supply of primary care physicians has been linked to numerous health outcomes for children, including overall access to care, rates of unnecessary hospitalizations, and local rates of infant mortality and low birth weight.^13^, ^14^, ^15^, ^16^ Additionally, researchers have found evidence that an increased supply of primary care practitioners is especially beneficial in areas with high levels of social disparities.^17^ The childhood health outcomes influenced by physician supply are associated with educational success in both direct and indirect ways. For example, lower birth weight has been directly correlated with reduced cognitive performance, and preventable hospitalizations for conditions like asthma have been associated with reduced academic achievement due to increased school absences.^18^, ^19^, ^20^, ^21^ In areas with low physician supply, families may be more likely to miss well‐child visits and other non‐emergency care, which can be critical to catching and treating early developmental delays and other conditions that can affect learning, such as impaired hearing and eyesight. With these links in mind, this paper explores potential associations between local physician supply and early academic achievement.

Filling this literature gap is important for understanding the feedback loop between education and health in the early years of life. Decades of research in child development have confirmed the importance of early childhood in shaping long‐term health outcomes, with education being one of the processes by which scholars, practitioners, and policymakers have sought to improve these outcomes.^22^, ^23^, ^24^, ^25^ Education is a social determinant of health and, conversely, a number of childhood health factors are linked to educational attainment.^26^, ^27^, ^28^, ^29^, ^30^, ^31^, ^32^ Despite this robust body of literature, we still have little understanding of how this feedback loop operates systemically. This limited understanding is, in part, a data problem: because of patient and student privacy protections, many of the measures relevant to child health are not available to schools, and many of the measures relevant to early learning are not available to pediatric providers. This is a dilemma with publicly available aggregate data as well, since interdisciplinary researchers are often limited to using county‐ or state‐level data in their analyses. With this paper, we partially address this data problem by creating a physician‐to‐child‐population ratio for every school district in the country. In so doing, we hope to highlight local child physician supply as an understudied feature of the child wellbeing landscape.

This study has two aims. Our first aim was to construct a unique measure of physician supply to investigate how child physicians (physicians trained as either pediatricians or family physicians) are distributed across U.S. school districts. This analysis provides the first description, to our knowledge, of how this important childhood health factor is distributed across educational environments and the student populations being served (or underserved) by these physicians.^33^, ^34^, ^35^ We incorporated both pediatricians and family physicians because family physicians commonly provide pediatric care in rural communities, though it is important to note that, on average, only 15% of visits to family physicians are from children.^35^, ^36^ Our second aim was to use population‐level data to describe associations between the national distribution of child physicians and local levels of early academic achievement.

## 2 METHODS

To analyze whether early academic achievement is associated with local child physician supply, we first outlined our conceptualization and operationalization of early academic achievement. We next identified our data sources for known correlates of academic achievement. We then developed a novel measure of local physician supply and linked our physician supply measure to our academic achievement measure and its correlates. We included a measure representing the local proportion of uninsured children in each school district, both for its role as a correlate of academic achievement and to compare it to our novel measure of physician supply.

### 2.1 Data sources

The primary data source for this paper is the Stanford Education Data Archive (SEDA, version 4.1), which uses nearly 430 million standardized test scores from all U.S. public school students in grades three to eight to construct measures of academic achievement for every community in the U.S. between academic years 2008–2009 and 2017–2018. Most state achievement tests are not directly comparable and often change over time. By linking assessment data drawn from the ED*Facts* database at the U.S. Department of Education to a common scale using the National Assessment of Educational Progress, SEDA enables comparisons of student achievement across grades, states, and years for the first time.^37^

### 2.2 Outcome variable

We used third‐grade test scores as our education achievement measure based on robust evidence linking health and educational achievement in early childhood.^25^, ^38^ Test scores were averaged across subjects (mathematics and English Language Arts) and academic years (2008–2009 through 2017–2018). To aid in interpretation of this measure, note that the average U.S. student's score improves by one‐third of a standard deviation (SD) per grade. Thus, a district where average test scores are 0.33 SD is performing roughly one grade level ahead of the national average for that grade. We interpret average third‐grade test scores as a measure of early educational opportunity. In other words, we consider these scores to be a reflection of the average child's opportunity to learn from their families, in their neighborhoods, from their peers, and in their childcare settings, as well as in their early elementary school years.^39^ Under this conceptualization, systemic differences in achievement are understood as opportunity gaps: they reflect inequalities in opportunities to learn undergirded by differential access and exposure to resources and stressors.^40^, ^41^

### 2.3 Covariates

As noted in the Introduction, most variation in student test scores is driven by sociodemographic factors and the structural inequalities linked to those factors, which we account for in the covariates noted below. School district‐specific qualities such as district enrollment size, student‐teacher ratio, and per pupil expenditure, to name a few, explain a small amount of variation in academic achievement beyond these factors, and as such, were not included in our analysis.

#### 2.3.1 Socioeconomic status

SEDA provides estimates of each district's average socioeconomic status using the National Center for Education Statistics (NCES) Education Demographic and Geographic Estimates (EDGE) program data, which tabulates American Community Survey (ACS) data within geographic school district boundaries. The ACS and EDGE data are reported as 5‐year averages and SEDA uses the 2005–2009 through 2014–2018 waves of EDGE data. The SES measure is constructed by taking the first principal component of six variables reported in the EDGE data: median family income, proportion of adults with a bachelor's degree or higher, household poverty rates, proportion of adults that are unemployed, proportion of households receiving SNAP benefits, and proportion of households with children that are headed by a single mother.

#### 2.3.2 Race/ethnicity

The district‐level racial/ethnic composition measure is derived from school‐level covariate data that is drawn from the Common Core of Data (CCD), which provides the racial/ethnic composition of students in each school.^37^

#### 2.3.3 Geographic type

We used SEDA's district‐level urban‐centric locale codes sourced from the CCD and created by NCES. This geographic indicator categorizes communities into four primary types—rural, town, suburban, and city—which are defined by proximity to densely populated areas rather than by specific municipal boundaries. Under this scheme, rural locales are census‐defined rural territories that are located outside of densely settled areas known as urbanized areas or urban clusters.^42^

#### 2.3.4 Insurance status

To construct the rate of uninsured children in each school district, we accessed ACS 5‐year district health insurance data for years 2009–2013 and 2014–2018.^43^ We restricted the sample for each file to the pediatric population (population under 18 and population under 19, respectively), producing a count of uninsured children in every district across the country. We divided this by the child population estimate in each respective file to produce the rate of uninsured children. Finally, we averaged the 2009–2013 and 2014–2018 insurance rates together to reduce sampling error and produce more reliable estimates. To account for the uncertainty in our insurance measure, particularly in districts with smaller populations, we generated precision‐weighted estimates for the district rate of uninsured children.

### 2.4 Predictor variable

We constructed a district‐level measure of child physician supply by creating a physician‐to‐child‐population ratio for every school district in our sample. We first generated a count of child physicians in every district by accessing the National Plan and Provider Enumeration System Downloadable File, which provides the practice location of every active physician in the United States.^44^ We used healthcare provider taxonomy codes to restrict physician observations to pediatricians and family physicians only, resulting in over 255,000 physicians whose addresses were then geocoded onto a geographic school district shapefile. Of note, medicine‐pediatrics residency graduates who primarily work with children are included in this analysis as pediatricians. The resulting data provides the count of child physicians who practice within the boundaries of every U.S. geographic school district. To convert this into a physician‐to‐child‐population ratio, we accessed child population estimates from two sets of ACS 5‐year data. This variable provides an estimate of the child population in every U.S. geographic school district through multi‐year sampling. We combined 2009–2013 data with 2014–2018 data to reduce sampling error and produce more reliable estimates. Using the district physician counts and district child population estimates, we generated a ratio representing the number of child physicians per 1000 children. We additionally constructed a ratio that uses pediatricians only, representing the number of pediatricians per 1000 children.

We utilized a few exclusion criteria to ensure we were using uniformly high‐quality data. We removed 47 observations with child population measures that are over 30% noise (coefficient of variance >30%). We excluded an additional 44 observations with a physician‐to‐child‐population ratio over 35 (>99th percentile) or a rate of uninsured children over 44% (>99th percentile). This resulted in a sample of 12,297 (99.6%) school districts.

### 2.5 Analysis

We conducted general descriptive statistics on the sample, including sociodemographic, education, and health data. We next examined the correlations between child physician supply and other variables, which allowed us to determine whether physician‐to‐child‐population ratios varied based on sociodemographic and structural features of school districts.

We next fit a set of multivariable regression models to estimate the associations between district test score outcomes and district‐level child physician supply while controlling for factors associated with test score outcomes, including community socioeconomic status, percentage of White students, and the percentage of uninsured children. In our national models, we included a fixed effect for the state, to eliminate any confounding introduced by unobservable state‐level characteristics.

We used this model across all districts in our sample, and again within subgroups of our data. Because we observed a nonlinear relationship between physician supply and third‐grade test scores in our descriptive statistics, we divided the sample into tertiles based on local physician supply. We did this to determine whether the size of the association between our predictor and outcome changed relative to the physician‐to‐child‐population ratio in a school district. In other words, we sought to observe whether the association was stronger or weaker in communities with higher or lower levels of physician supply.

We also utilized the nonlinear approach of using B‐splines, which are non‐parametric and allow for nonlinear treatment of continuous predictors, such as physician supply, in regression models that contain other variables. We prefer them to alternatives, such as inserting squared and/or cubic terms, given the extreme behavior for values far from the mean that such approaches imply. We first mapped physician supply to the five splines that cumulatively allow for differential response behavior in the outcome at different levels of physician supply. We then included those five splines in subsequent regression analyses in place of physician‐to‐child‐population ratio. Rather than focus on regression estimates related to the spline predictors, we used the resulting coefficient estimates to predict achievement for various levels of physician supply when we hold other covariates constant (at the mean value for each covariate); these results are presented graphically.

## 3 RESULTS

### 3.1 The distribution of child physicians

Over 255,000 pediatricians and family physicians were distributed across 8658 (70.4%) of the 12,297 school districts included in this sample, meaning 3639 (29.6%) districts had no child physician. Looking only at pediatricians, over 80,000 pediatricians were distributed across just 4333 (35.2%) of 12,297 school districts. In other words, two‐thirds of U.S. school districts, which collectively serve over 640,480 (17.7%) of roughly 3.6 million students per grade, had no pediatrician within their boundaries. Overall, the average school district had 2.51 child physicians per 1000 children, but only 0.43 pediatricians per 1000 children (Table 1).

**TABLE 1:** Descriptive statistics of school districts, stratified by geographic Type and physician supply.

	Mean	SD	Min	Max
All school districts (*n* = 12,297)				
Pediatrician‐to‐child‐population ratio	0.43	1.09	0.00	25.10
Physician‐to‐child‐population ratio	2.51	3.09	0.00	33.46
Rate of uninsured children	6.65	5.60	0.00	43.98
Third grade achievement	0.01	0.34	−2.44	1.66
Socioeconomic status	0.33	0.85	−4.40	2.91
Percent White	73.68	27.21	0.00	100.00
Rural districts (*n* = 6421)				
Pediatrician‐to‐child‐population ratio	0.11	0.58	0.00	20.94
Physician‐to‐child‐population ratio	1.65	2.71	0.00	30.30
Rate of uninsured children	7.75	6.42	0.00	43.98
Third grade achievement	−0.02	0.32	−2.44	1.66
Socioeconomic status	0.29	0.71	−4.12	2.63
Percent White	80.64	23.75	0.00	100.00
Non‐rural districts (*n* = 5876)				
Pediatrician‐to‐child‐population ratio	0.79	1.38	0.00	25.10
Physician‐to‐child‐population ratio	3.44	3.20	0.00	33.46
Rate of uninsured children	5.45	4.24	0.00	36.49
Third grade achievement	0.06	0.36	−1.56	1.15
Socioeconomic status	0.37	0.97	−4.40	2.91
Percent White	66.07	28.69	0.00	99.61
Tertile 1: Low supply (*n* = 4099)				
Physician‐to‐child‐population ratio	0.05	0.14	0.00	0.60
Rate of uninsured children	7.78	6.58	0.00	43.98
Third grade achievement	−0.05	0.35	−1.74	1.66
Socioeconomic status	0.36	0.72	−4.12	2.89
Percent White	76.89	26.87	0.00	100.00
Tertile 2 (*n* = 4099)				
Physician‐to‐child‐population ratio	1.75	0.65	0.60	2.93
Rate of uninsured children	6.27	5.18	0.00	43.87
Third grade achievement	0.02	0.34	−1.52	1.15
Socioeconomic status	0.33	0.92	−3.92	2.91
Percent White	70.76	29.08	0.02	99.99
Tertile 3: High supply (*n* = 4099)				
Physician‐to‐child‐population ratio	5.73	3.33	2.93	33.46
Rate of uninsured children	5.90	4.71	0.00	40.27
Third grade achievement	0.07	0.33	−2.44	1.13
Socioeconomic status	0.31	0.88	−4.40	2.72
Percent White	73.37	25.20	0.10	100.00

*Note*: SD refers to standard deviation. We tested whether means of distributions between rural and nonrural and between tertiles were equivalent. *p*‐values for these tests were less than 1e‐10 for all comparisons except for the SES comparison among tertiles, which had a *p*‐value of 0.020.

Child physician supply was negatively correlated with rural district status (*r* = −0.29, *p* < 0.001), indicating that rural districts disproportionately have fewer child physicians than non‐rural districts (Table 2). We also found that child physician supply was weakly correlated with the rate of uninsured children (*r* = −0.083, *p* < 0.001), the percentage of White students (*r* = −0.018, *p* < 0.01), and (among pediatricians) socioeconomic status (*r* = 0.093, *p* < 0.001), although the correlations were weaker than those between other known sociodemographic factors, such as the correlation between socioeconomic status and the rate of uninsured children (*r* = −0.363, *p* < 0.001).

**TABLE 2:** Bivariate correlations between variables.

	(1)	(2)	(3)	(4)	(5)	(6)	(7)
(1) Physician‐to‐child‐population ratio	1.000						
(2) Pediatrician‐to‐child‐population ratio	0.580***	1.000					
(3) Third grade achievement	0.125***	0.145***	1.000				
(4) Rate of uninsured children	−0.083***	−0.123***	−0.363***	1.000			
(5) Socioeconomic status	−0.007	0.093***	0.685***	−0.363***	1.000		
(6) Percent White	−0.018**	−0.122***	0.518***	−0.333***	0.431***	1.000	
(7) Rural district	−0.289***	−0.307***	−0.116***	0.147***	−0.047***	0.268***	1.000

*p < 0.05; **p < 0.01; ***p < 0.001.

Table 1 shows that child physicians were overrepresented in non‐rural school districts compared to rural school districts by more than a 2–1 margin: the average rural district had 1.65 doctors per 1000 children compared to 3.44 doctors in non‐rural districts. In fact, over 49% of rural districts had no child physicians, while only 8% of non‐rural districts had no child physicians. These patterns were more dramatic among pediatricians, who were overrepresented in non‐rural districts by more than a 7–1 margin: almost 90% of rural districts had no pediatrician within its boundaries, compared to 38% of non‐rural districts. Rural students had less access to child physicians, and this was particularly true for rural places with large non‐White populations (Figure S1).

### 3.2 Associations between district‐level child physician supply and academic achievement

In unadjusted models, an increase of one child physician per 1000 children was associated with a 0.014 SD (95% CI, 0.012–0.016) increase in third‐grade test scores—roughly equivalent to 4% of a grade level (Table 3, column 1). Controlling for known correlates of academic achievement did not alter this association; the relationship between child physician supply and academic achievement remained at 0.014 SD (Table 3, column 2). Adding a state fixed effect to the model attenuated the relationship to 0.012 SD (Table 3, column 3). The relationship between pediatrician physician supply and academic achievement was 225% larger than that of child physicians overall: an increase of one pediatrician per 1000 children was associated with a 0.027 SD (95% CI, 0.024–0.031) increase in third‐grade test scores—roughly 8% of a grade level.

**TABLE 3:** Associations between child physician supply and third grade achievement.

	Child physicians (pediatricians + family physicians)			Pediatricians
	Simple	Add covariates	Full	Full
Physician‐to‐child‐population ratio	0.014 (0.001)***	0.014 (0.001)***	0.012 (0.001)***	
Pediatrician‐to‐child‐population ratio				0.027 (0.002)***
Rate of uninsured children		−0.007 (0.001)***	−0.006 (0.001)***	−0.006 (0.001)***
Socioeconomic status		0.223 (0.003)***	0.228 (0.003)***	0.223 (0.003)***
Percent White		0.003 (0.000)***	0.002 (0.000)***	0.002 (0.000)***
State fixed effects			Yes	Yes
Constant	−0.021 (0.004)***	−0.096 (0.003)***	−0.091 (0.003)***	−0.072 (0.002)***
*N*	12,297	12,297	12,297	12,297
*R* ^2^	0.02	0.55	0.50	0.50

*Note*: Standard errors listed in parentheses. Rate of uninsured children and percentage of White students are mean centered.

*p < 0.05; **p < 0.01; ***p < 0.001.

The associations were highly heterogeneous: the effect sizes were much larger in districts with relatively low supplies of child physicians. In the highest tertile (average 5.73 child physicians per 1000 children), mean third‐grade test scores were 0.07 SD, 235% higher than the mean test score of −0.05 SD in the lowest tertile (average 0.46 physicians per 1000 children) *despite* the slightly higher socioeconomic status in the low supply districts (Table 1). After running our model, we found that one additional child physician in a district with high supply was associated with an increase of 0.004 SDs (95% CI, 0.0018–0.0053), whereas in low supply districts, an additional child physician was associated with an increase of 0.163 SDs (95% CI, 0.108–0.219), roughly equivalent to 90 additional days of learning, or an additional half of a grade level of achievement (Table 4). There was an increase of 4000% in the effect size between the lowest and highest tertiles.

**TABLE 4:** Associations between physician supply and third grade achievement, by tertile.

	1st tertile	2nd tertile	3rd tertile
Physician‐to‐child‐population ratio	0.163 (0.028)***	0.025 (0.004)***	0.004 (0.001)***
Rate of uninsured children	−0.003 (0.002)*	−0.005 (0.001)***	−0.011 (0.001)***
Socioeconomic status	0.192 (0.007)***	0.230 (0.004)***	0.242 (0.004)***
Percent White	0.003 (0.000)***	0.002 (0.000)***	0.002 (0.000)***
State fixed effects	Yes	Yes	Yes
Constant	−0.136 (0.005)***	−0.091 (0.008)***	−0.029 (0.006)***
*N*	4099	4099	4099
*R* ^2^	0.34	0.60	0.58

*Note*: Standard errors listed in parentheses. Rate of uninsured children and percentage of White students are mean centered.

*p < 0.05; **p < 0.01; ***p < 0.001.

**FIGURE 1:**
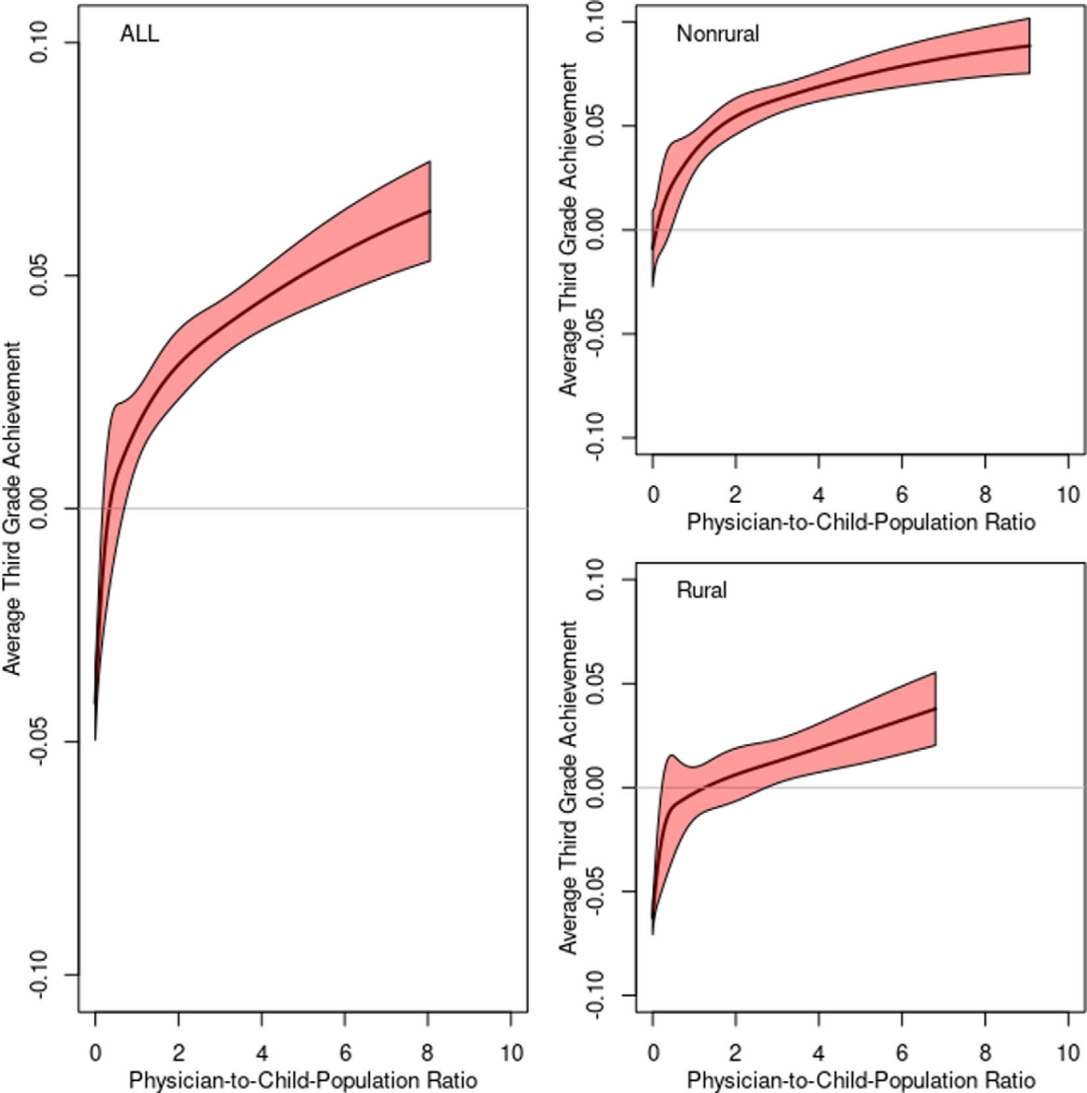
Projected levels of achievement as a function of physician‐to‐child‐population ratio using B‐splines. [Color figure can be viewed at wileyonlinelibrary.com]

We also used splines to allow for nonlinearity of the association between physician‐to‐child‐population ratio and achievement (Figure 1). The increase in academic achievement associated with higher physician supply was rapid in the low‐supply regions. In contrast, gains were much more modest when physician‐to‐child‐population ratio was larger. These findings held in both rural and non‐rural settings.

Because higher physician supply is associated with higher numbers of residency slots in a state—and because these residency slots are often concentrated in urban areas—we conducted a sensitivity check by removing the 25 most populated districts from our model.^34^ We found that the results were nearly identical to our original model.

## 4 DISCUSSION

We find evidence that the distribution of child physicians is associated with early academic achievement. Children in districts with more child physicians—and especially districts with more pediatricians—do better in school as measured by achievement tests in third grade. This relationship operates independently of community socioeconomic status and racial/ethnic composition. An additional child physician per 1000 children is associated with a 0.012 SD increase in achievement, or roughly a week of each school year, while an additional pediatrician is associated with nearly a 0.03 SD increase in achievement, which is roughly 2 weeks of each school year.

These associations vary in critical ways. The relationship between physician supply and test scores is strongest in places with low levels of physician supply, where the presence of one additional child physician is associated with a half of a grade level of higher achievement—or about 90 extra school days of learning. In the context of education research, this is a substantial difference, and is particularly notable given the magnitude of the effect and the fact that local physician supply is not a traditional educational intervention.^45^ We believe this finding elucidates one potential pathway by which children's early health environments contribute to early academic achievement, especially in districts with few pediatricians and family physicians.

Given the heterogeneity in this relationship, it is important to note that the distribution of child physicians is highly unequal in the United States. Rural students, especially rural students of color, have particularly low access to child physicians. Shockingly, nearly 90% of rural school districts have no pediatrician within their boundaries, and 50% have no child physician at all, highlighting the disparate access to pediatric care experienced by rural children. These patterns align with overall trends in access to healthcare in rural areas, which are often designated as physician shortage areas by the federal government, and increasingly suffer from hospital closings.^46^, ^47^

This paper finds compelling evidence that students with lower levels of early academic achievement tend to be the same students that have low levels of access to nearby pediatric care. Furthermore, we would like to emphasize that regardless of whether the observed association is causal, it is of grave concern that children throughout the U.S. systemically face barriers to access and success in two sectors that are deeply intertwined with child wellbeing and outcomes across the life course. It is imperative that researchers, practitioners, and policymakers engage in cross‐sector collaboration to remove these barriers and create more equitable access to opportunities for all children.

This study has several limitations. First, this study cannot explain the mechanisms underlying the relationship we see between child physician supply and achievement. Though we outline several pathways through which this association may be operating, it is important to note that more research must be conducted to rule out potential confounders and explore how the local supply of pediatric advanced practice practitioners, local hospital capacities, and regional patterns of physician use may be playing a role in the described relationship. School‐based health centers, which are gaining momentum in the push to meet children's basic needs in service of better educational outcomes, should be factored in future analyses as well. Perhaps most importantly, more work could be done to explore the utility of local physician‐to‐child‐population ratios, ideally by utilizing data that links student test scores with the utilization of pediatric care at the individual level. Second, some geographic school districts are quite small, and there are a number of feasible scenarios in which families may utilize pediatric care outside of their school district—particularly in small suburban and rural districts that are relatively close to more densely populated areas. Still, health care researchers have wrestled with the fact that there is no obvious unit of geography for health care, especially when seeking to understand the health landscape at the sub‐county level.^48^ Because child wellbeing is fundamentally influenced by the health and education landscape in which they live, a district‐level measure of physician supply is a potentially important measure for education and health researchers seeking to understand the intersection of these systems. Third, the achievement data represents grade three, so we cannot speak to levels of achievement in earlier grades, which may differ from what we observe.

Although the cross‐sectional nature of the data precludes causal inferences, we nonetheless believe the findings are important and can focus attention on a potentially significant feature of the child development landscape that may be amenable to policy change. Further investigation of this relationship could provide evidence to support the growth and redistribution of the child physician workforce in order to achieve benefits for children that extend beyond the realm of health. Since physician training is publicly funded, policymakers should develop, improve, and monitor policies aimed at distributing child physicians in a more equitable way if communities are reaping uneven benefits from taxpayers' contributions to the U.S. medical workforce. For example, expanding medical student loan forgiveness may be an effective way to achieve this redistribution, since research has demonstrated that physicians with more education debt are less likely to serve in health professional shortage areas.^49^

Patterns in early academic achievement vary greatly across communities in the United States, and most research seeking to understand this variation has overlooked the role of the local health environment as a potential contributing factor. This gap exists despite the fact that researchers have made great advances in our understanding of the complex interplay between early childhood health, brain development, and learning—particularly their roles in influencing life outcomes for all children. This paper contributes to our understanding of the feedback loop between education and health during early childhood by detailing the relationship between local child physician supply and early academic achievement at a level of detail not previously possible. The local supply of child physicians in a district merits further attention as a novel feature of the landscape relevant to child development and early academic achievement.

## Supporting information


**Figure S1.** Unadjusted Relationship Between Physician‐to‐Child‐Population Ratio and District Sociodemographic Characteristics: Socioeconomic Status (left) and Percentage of White Students (right).
Note: Rural districts and non‐rural districts are each sorted into 100 equal‐sized bins.
