# Supplementary material for: The distribution of child physicians and early academic achievement
Source: Health Serv Res. 2023 Jun 7;58(Suppl 2):165–74. doi: 10.1111/1475-6773.14188 (PMC10339172; doi:10.1111/1475-6773.14188)
Supplement: Supplementary file 1 — Figure S1. Unadjusted Relationship Between Physician‐to‐Child‐Population Ratio and District Sociodemographic Characteristics: Socioeconomic Status (left) and Percentage of White Students (right). Note: Rural districts and non‐rural districts are each sorted into 100 equal‐sized bins. [file HESR-58-165-s001.docx]

**Supplemental Information**

**Figure S1.** Unadjusted Relationship Between Physician-to-Child-Population Ratio and District Sociodemographic Characteristics: Socioeconomic Status (left) & Percentage of White Students (right).


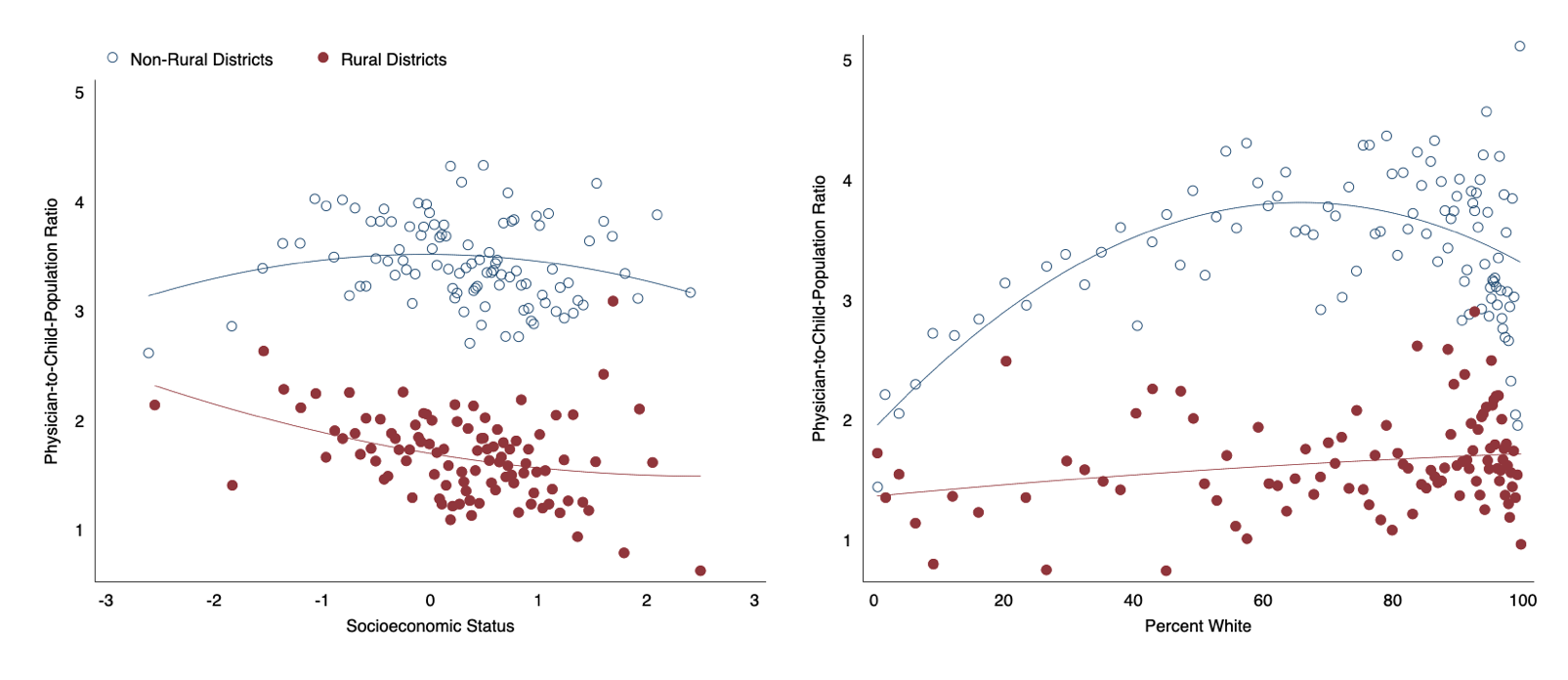


Note: Rural districts and non-rural districts are each sorted into 100 equal-sized bins.
